# MRI-assessed end-organ damage in hypertension: association between aortic stiffness, cardiac function and lacunar brain infarcts

**DOI:** 10.1186/1532-429X-11-S1-P23

**Published:** 2009-01-28

**Authors:** Anne Brandts, Saskia GC van Elderen, Jos JM Westenberg, Jeroen van der Grond, Mark A van Buchem, Menno V Huisman, Lucia JM Kroft, Jouke T Tamsma, Albert de Roos

**Affiliations:** Leiden University Medical Center, Leiden, Netherlands

**Keywords:** Magnetic Resonance Imaging, Hypertensive Patient, Aortic Arch, Pulse Wave Velocity, Left Ventricular Mass

## Introduction

Hypertension is a major risk factor for cardiovascular morbidity and mortality through its effects on target organs including the heart, the blood vessels and the brain. Early detection of cardiac and cerebral outcome, before irreversible damage has occurred, can contribute to further decline in hypertension-related death.

Pulse Wave Velocity (PWV), defined as the propagation speed of the systolic pressure or flow wave through the aorta, is a surrogate marker for aortic stiffness. PWV has been shown to be an independent predictor of stroke and cardiac mortality in hypertensive patients. Gold standard for PWV-assessment comes from intravascular pressure measurement but this requires an invasive procedure, not suitable for screening. The current method-of-choice is non-invasive echo Doppler. Magnetic resonance imaging (MRI) provides an alternative, with the advantage over echo Doppler that image acquisition does not have a limited acoustic window and the aorta can be visualized along its full length, allowing for more accurate assessment of the path lengths of pulse waves. Also, MRI is the gold standard for assessment of cardiac left ventricular (LV) function and mass and cerebral lacunar infarcts and white matter hyperintensities (WMHs). An integrated MRI-approach for the assessment of aortic stiffness, cardiac function and brain examination is performed in hypertensive patients. Our hypothesis is that early end-organ damage (i.e. increased aortic stiffness, decreased cardiac function and cerebral lacunar infarcts and WMHs) are interrelated in hypertensive patients.

## Purpose

To assess the association between aortic PWV, cardiac LV function and mass as well as cerebral lacunar infarcts and WMHs in hypertensive patients using MRI.

## Methods

**Figure 1:**
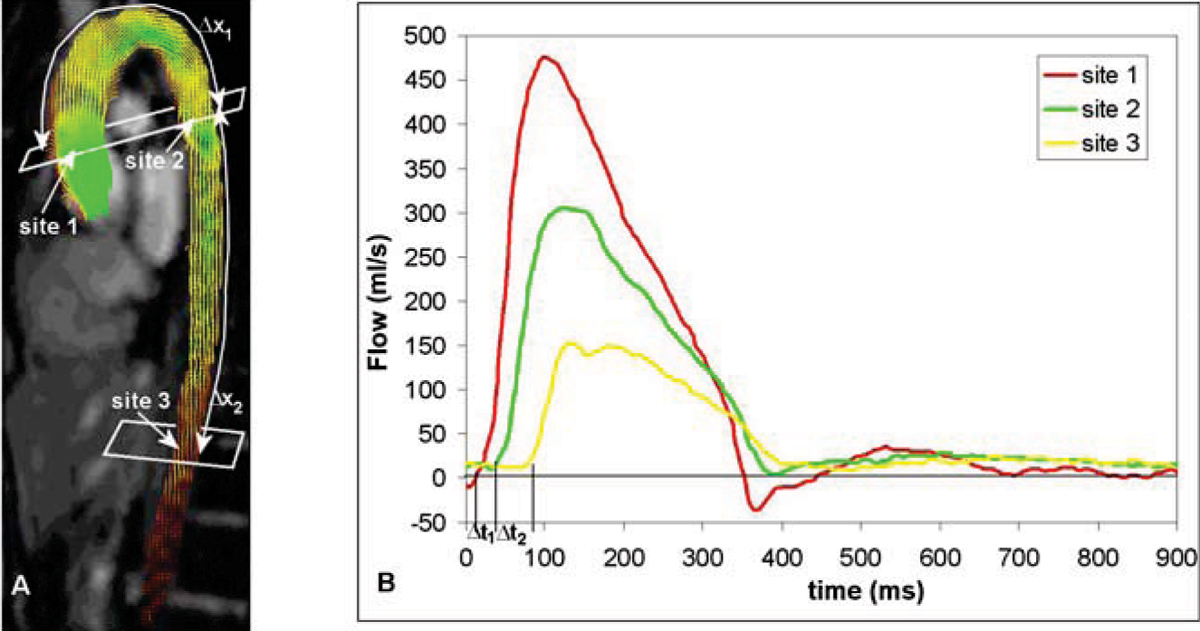
**PWV determination**. A, a sagittal gradient-echo image of the aorta is shown. Velocity-encoded MRI is performed perpendicular to the aorta at two levels: site one is positioned at the level of the pulmonary trunk; site two is positioned at the distal descending abdominal aorta 7.5 cm below the diaphragm; Δx_1_: path length of the aortic arch Δx_2_: path length of the descending aorta. B, the flow curves of the ascending, proximal and distal descending aorta are shown; Δt_1_ and Δt_2_ denote the transit-time between the arrival of the pulse wave at subsequent measurement sites. Aortic PWV is defined as Δx/Δt (m/s).

MRI of the aorta, heart and brain was performed in 50 consecutive hypertensive patients (19 male, 31 female; mean age 49 ± 12 years). Aortic PWV was determined from one-directional through-plane velocity-encoded (VE) MRI at the ascending aorta, proximal and distal descending aorta (Figure 1). From VE MRI, flow graphs were obtained. High temporal resolution (6–10 ms) allows for accurate assessment of the transit-time between the arrival of the pulse wave at subsequent measurement sites. A value for PWV was obtained for the aortic arch and the descending aorta.

Short-axis MRI using steady-state free precession was used to measure LV systolic function and mass. An ECG-gated gradient-echo sequence with VE was performed to measure blood flow across the mitral valve to determine LV diastolic function. For detection of lacunar infarcts and WMHs, patients underwent T2 and Fluid Attenuated Inversion Recovery (FLAIR) acquisitions.

The association between aortic PWV, cardiac and cerebral parameters was calculated using univariate and multivariate linear and logistic regression analyses.

## Results

Mean systolic BP was 152 ± 22 mmHg, mean diastolic BP 88 ± 13 mmHg. Mean aortic arch PWV was 7.3 ± 2.5 m/s, mean descending aorta PWV 6.9 ± 2.7 m/s.

After adjustment for age, gender and hypertension duration, aortic PWV was shown to be independently associated with LV mass (r = 0.41, p = 0.03) and lacunar brain infarcts (OR = 1.8, 95% CI 1.0–3.0, p = 0.02) and not with LV function and WMHs.

## Conclusion

Aortic PWV is independently associated with LV mass and lacunar brain infarcts, both indicators of early end-organ damage of hypertension.

